# Heat stress and the velocity–duration relationship in amateur runners

**DOI:** 10.14814/phy2.70013

**Published:** 2024-08-16

**Authors:** Eric Leslie, Jyotika Erram, Daniel T. Cannon

**Affiliations:** ^1^ School of Exercise & Nutritional Sciences San Diego State University San Diego California USA

**Keywords:** exercise, heat‐stress response, running, thermoregulation

## Abstract

Tolerance to high‐intensity constant power exercise can be characterized by the hyperbolic power‐duration (or velocity–duration) relationship. The hyperbola is defined by the asymptote (critical power or velocity) and the curvature constant (*W*′ or *D*′). The effects of thermoregulatory stress on middle‐distance running performance are equivocal—possibly due to the complexities of the hyperbolic velocity–duration relationship for these relatively short duration events. We aimed to measure the effects of heat stress on the velocity–duration relationship in amateur runners. Fifteen participants (23 ± 6 years) completed a series of constant‐velocity running bouts to intolerance in three heat indices (MILD: 20°C, VERY HOT: 38°C, EXTREME: 55°C). Critical velocity (CV) in MILD (3.52 ± 0.86 m/s) was higher than VERY HOT (3.39 ± 0.82 m/s) and EXTREME (3.29 ± 1.05 m/s; F[2.28] = 3.80, *p <* 0.035) with no effect of thermal stress on *D*′ (F[2.28] = 2.48, *p =* 0.11). In amateur competitive/recreational runners, heat stress of ≥38°C heat index negatively affected CV. Thus, even during relatively short events, such as middle‐distance running where fluid loss is not a primary concern, heat stress may negatively impact performance.

## 1 INTRODUCTION

Tolerance to high‐intensity running is characterized by a hyperbolic velocity–duration relationship (Hill, 1925; Poole et al., 2016). The relationship is defined by two parameters—the curvature constant, termed *D*′, and an asymptote, critical velocity (CV). CV (or power when appropriate) demarcates sustainable and predictably unsustainable exercise. Velocities above CV demand nonsteady state metabolism in which the rate of O_2_ consumption (V̇O_2_) is drawn to the maximum. During this time, a substantial contribution from nonoxidative and finite energy reserves is required to provide ATP for the demands of the task. These disturbances in homeostasis elicit predictable exercise intolerance due to accumulation of fatigue‐related metabolites and attainment of aerobic systems limits (Grassi et al., 2015; Poole et al., 2016).

Heat stress effects on CV (or power) are also somewhat equivocal. Others report either no effects or a modest reduction in the estimated CV, and so far no changes in the curvature constant (Kaiser et al., 2021; Kuo et al., 2021). These heat stress studies on critical power have used the 3‐min all‐out test (Vanhatalo et al., 2007, 2008) rather than multiple constant power bouts to characterize the power–duration relationship.

If exercise tolerance is disrupted due to environmental stressors such as hypoxia or heat stress, the apparent effect size is considerably larger for longer duration tasks (Lafrenz et al., 2008). Not only is this due to the shape of the power–duration relationship, but also the simple exposure requirement for heat stress affecting performance (Richard & Koehle, 2022). For example, hot and humid conditions negatively affect endurance running performance but may favor sprint performance (Ball et al., 1999; Lafrenz et al., 2008; Periard et al., 2011; Sargeant, 1987; Viveiros et al., 2012). Conversely, the effects on middle distance performance are equivocal (Guy et al., 2015). The uncertainty about heat stress and middle distance running is likely just a result of the shape of the velocity–duration relationship and the duration of these events. Changes in exercise tolerance are nonlinearly related to the duration of the exercise (or event distance). If event duration is the primary outcome variable and CV and the curvature constant are not measured, then the effects of any environmental stress on exercise tolerance will be obscured.

To address some of these roadblocks to understanding heat stress during supra‐critical power performance, we aimed to measure the effects of heat stress on the velocity–duration relationship in using amateur competitive/recreational runners. We hypothesized that CV would be negatively affected by heat stress in these runners.

## 2 MATERIALS AND METHODS

### 2.1 Participants

Volunteers were men and women 18–45 years that regularly participated in amateur competitive and recreational running. Participants were considered amateur competitive or recreational runners if they were not sponsored runners and they had no elite racing experience. Fifteen healthy runners (11 men, four women; 23 ± 6 years, 18–40 years; 1.76 ± 0.07 m; 68.9 ± 11.3 kg) met the criteria and volunteered to participate. Volunteers provided written informed consent and were screened for pregnancy with a commercial urine test kit (Fisher Healthcare Sure‐Vue Urine hCG, Cat. 23–900‐527), and cardiovascular risks with the Physical Activity Readiness Questionnaire (PAR‐Q) prior to beginning the study. The San Diego State University Institutional Review Board approved the protocol.

### 2.2 Experimental design

Participants completed four constant‐velocity running bouts in each of the three levels of heat stress. Each bout of exercise was supra‐CV and completed to the limit of tolerance. Heat stresses included MILD (heat index [HI] = 20°C; 21°C with 40%RH), VERY HOT (HI = 38°C; 34°C with 50% RH), and EXTREME (HI = 55°C; 40°C with 50%RH) conditions. The National Weather Service (NWS) HI equation was used to calculate heat indices based on temperature and humidity. The NWS heat index equation is a multiple regression analysis that has assumed magnitudes for an extensive number of parameters that affect thermal stress when parameters such as wind speed, cloud cover, and sun angle are unavailable, leaving temperature and humidity as the only inputted variables (Rothfusz, 1990; Steadman, 1979). This equation has been used in national and international environmental research to accurately calculate HI (Anderson et al., 2013). Table 1 provides the calculations used for HI.

**TABLE 1:** National Weather Service multiple regression heat index equation.

Calculation	Criteria	Equation
Full Regression	HI ≥26.7°C/80°F	HI = −42.379 + 2.04901523*T + 10.14333127*RH − 0.22475541*T*RH − 0.00683783*T*T − 0.05481717*RH*RH + 0.00122874*T*T*RH + 0.00085282*T*RH*RH − 0.00000199*T*T*RH*RH
Adjustment 1	Subtracted if T = 80–122°F and RH <13%	[(13 − RH)/4]*SQRT{[17 − ABS(T − 95.)]/17}
Adjustment 2	Added if T = 80–87°F and RH >85%	[(RH − 85)/10]*[(87 − T)/5]
Simplified Regression	HI <26.7°C/80°F	HI = 0.5*{T + 61.0 + [(T − 68.0)*1.2] + (RH*0.094)}

Abbreviations: HI, heat index; RH, relative humidity (%); T, temperature (°F).

Heat stress conditions were chosen to incorporate HI ranges common for many athletic events (MILD), typically high HI observed during competitive summer athletic events (VERY HOT), and the lowest end of heat stress categorized as extreme danger by the NWS (EXTREME). Testing was conducted on six nonconsecutive days spread over 3 weeks with each test separated by ≥48 h. Participants were instructed to arrive at their normal hydration state prior to exercise, eat a light meal 2–3 h prior to the laboratory visit, and to refrain from exercise and alcohol for 24 h preceding the visit. For each day, participants completed two of the running bouts randomly chosen. The bouts were separated by 45 min of resting recovery in a room outside of the environmental chamber kept at 20°C. Participants were allowed water ad libitum during this recovery period.

### 2.3 Exercise testing

Participants acclimated for 10 min to the randomized heat stress condition (MILD: 20°C, VERY HOT: 38°C, EXTREME: 55°C) for each randomized constant‐velocity running bout. Following the 10 min seated period, volunteers completed a 5 min warm‐up at 2.2 m/s followed by an additional 5 min rest period. Participants then completed a constant‐velocity running bout on the treadmill to intolerance. The bouts were designed to bring about intolerance in ~2–15 min to characterize the velocity–duration relationship. A best estimate was used to set a velocity that was not sustainable for each participant. Following their performance of the bout, we used an iterative process to lengthen and shorten the bouts to adequately characterize the velocity–duration relationship. This naturally results in variable bout durations across the sample. Each participant would complete the same four velocity bouts across the heat stress conditions in a counterbalanced order.

Following intolerance, participants completed a 5 min cool‐down at 2.2 m/s. After a 45 min recovery period outside of the environmental chamber (20°C), participants repeated all steps for the second running bout. To ensure focus remained solely on running to intolerance, participants were blinded to the velocity, duration elapsed, and eventual tolerance time. Naturally, blinding is not possible for heat stress.

Running bouts were terminated at the limit of tolerance—defined as being unable to continue despite strong verbal encouragement. Participants had access to the treadmill emergency stop button. At the limit of tolerance, participants terminated the test themselves, signaled the researcher to stop the test, or the researcher stopped the test for safety reasons. Tympanic temperatures were measured continuously for safety. Bouts would have prematurely halted if tympanic temperature was >39.5°C, however this never occurred.

All testing was done on a custom built, calibrated treadmill set at 1% gradient (Vacumed, Ventura, CA) inside the temperature and humidity‐controlled environmental chamber (Nor‐Lake Scientific, Hudson, WI). The laboratory elevation was 135 m above mean sea level. Tympanic temperatures were measured throughout and recorded to the nearest 0.01°C (Adult Tympanic Temperature Sensor, 400 series thermistor, Starboard Medical, Yorba Linda, CA). ∆Temp was obtained from the difference tympanic temperatures recorded before and after all running bouts, resulting in ∆Temp for MILD, VERY HOT, and EXTREME. ∆Temp were included for statistical analysis if end‐test temperature stabilized and was recorded within 1 min of test termination. Borg's (6–20) rating of perceived exertion (RPE) was also recorded for each running bout. RPE was measured at the end of each bout was pooled based on the heat stress (*n* = 60 for MILD, VERY HOT, and EXTREME).

For each participant, velocity (m/s) and tolerable duration (s) were used to establish the hyperbolic curvature constant and asymptote within each level of heat stress:

(1) $$D^{\prime }=t\left(V-\mathrm{CV}\right)$$

where *D′* is the curvature constant, *t* is tolerable duration, *V* is running velocity and *CV* is the critical velocity asymptote. For simplicity, the CV and D*′* parameters were determined from linear regression by plotting *V* as a function of (*1/t*):

(2) $$V=D^{\prime }\left(1/t\right)+\mathrm{CV}$$

Data were analyzed with a one‐way RM ANOVA to test the effect of heat stress on CV, *D*′, ∆Temp, and Friedman test for ratings of perceived exertion (RPE) (GraphPad Prism 10, San Diego, CA). In the case of an omnibus test resulting in a rejection of the null hypothesis, we used *t*‐tests post hoc. Data are presented as mean ± SD.

## 3 RESULTS

**FIGURE 1:**
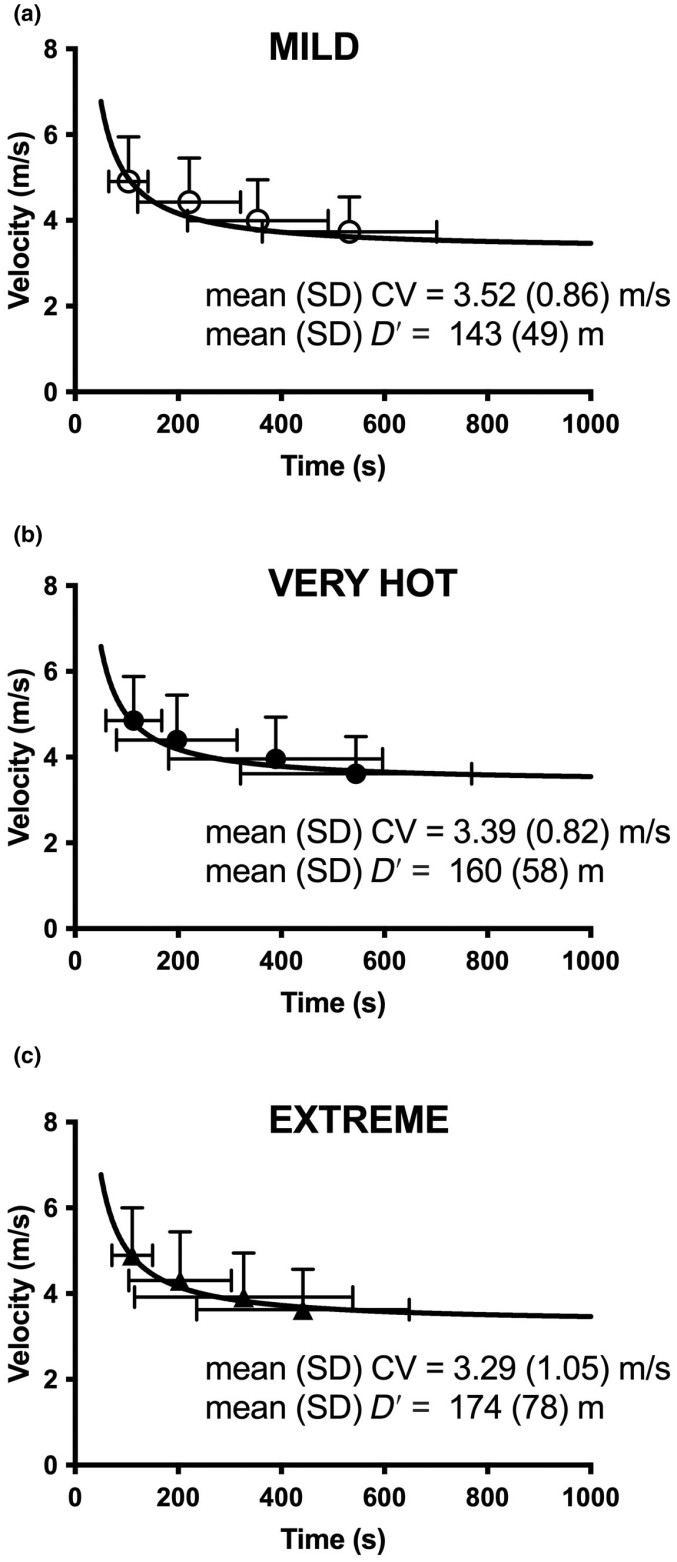
Velocity–duration relationships for amateur competitive/recreational runners. (a) Data included for MILD at a heat index (HI) of 20°C (*n* = 15). (b) Data included for VERY HOT at a HI of 38°C (*n* = 15). (c) Data included for EXTREME at a HI of 55°C (*n* = 15).

**FIGURE 2:**
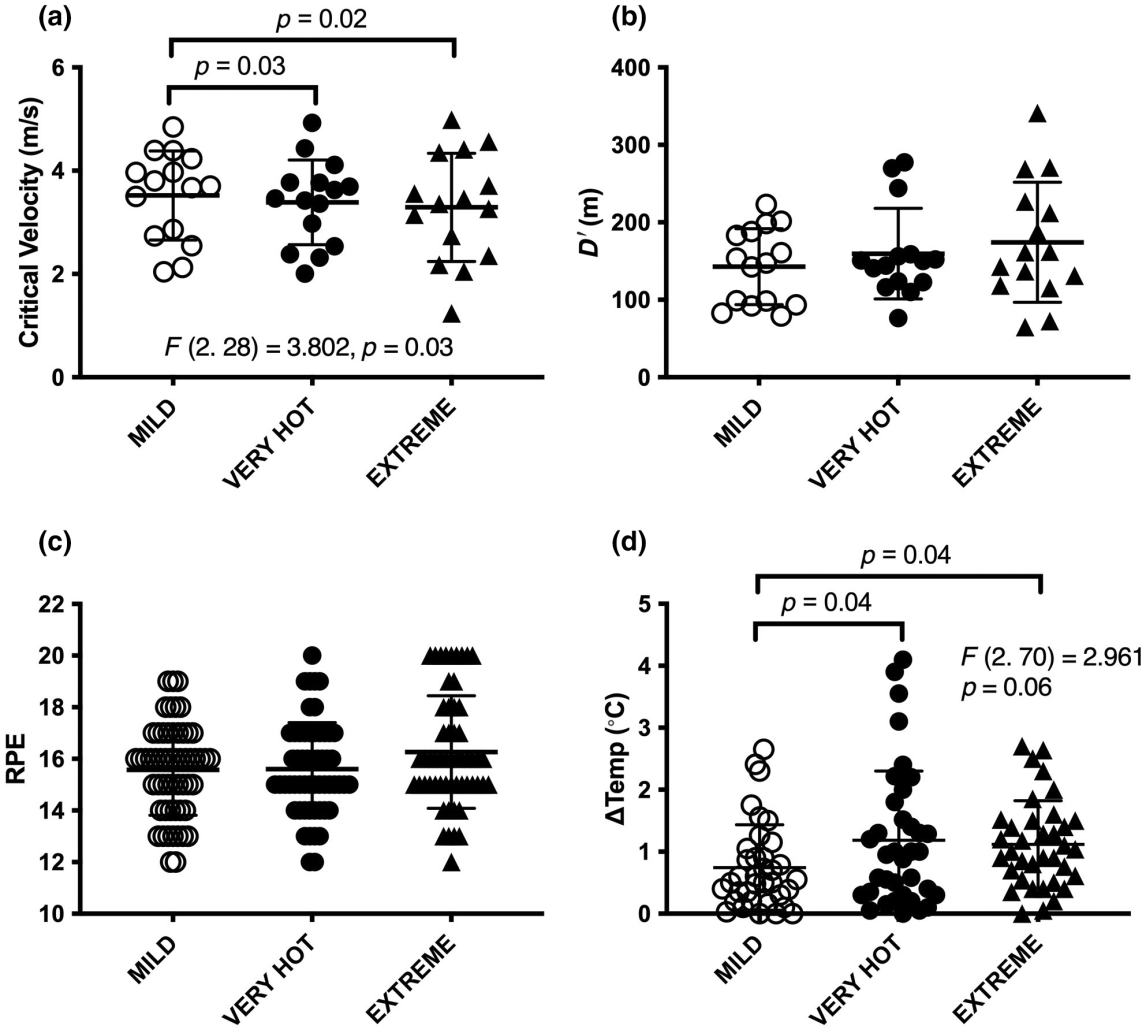
Individual and mean(SD) for critical velocity, curvature constant, RPE, and ∆Temp for amateur competitive/recreational runners. Data shown for MILD 20°C, VERY HOT 38°C, and EXTREME 55°C (a) Critical velocity. (b) Curvature constant (*D*′). (c) RPE measured before and after each constant velocity bout. (d) ∆Tympanic temp measured before and after each constant velocity bout.

The mean CV in MILD (3.52 ± 0.86 m/s) was greater than VERY HOT (3.39 ± 0.82 m/s), and EXTREME (3.29 ± 1.05 m/s; F[2.28] = 3.80; *p <* 0.035; *η* ^2^ = 0.15; Figure 1, Figure 2a) while no differences were found in *D*′ between the heat stress levels (*F*[2.28] = 2.48; *p* = 0.11; *η* ^2^ = 0.15; Figure 2d). Post hoc *t* tests revealed reductions in mean CV in VERY HOT compared to MILD (mean difference −0.13 ± 0.2 m/s; CI_Difference_ −0.25 to −0.02; *t*[14] = 2.45; *p* < 0.028) and in EXTREME compared to MILD (−0.23 ± 0.34 m/s; CI_Difference_ −0.42 to −0.04; *t*[14] = 2.58; *p* < 0.022).

RPE was not different across the heat stress conditions (Friedman = 3.64, *p* > 0.16; Figure 2c).

∆Temp increased with heat stress (*F*[2.70] = 2.96; *p* < 0.059; *η* ^2^ = 0.078; Figure 2d). Post hoc *t* tests revealed MILD (0.75 ± 0.69°C) different to VERY HOT (1.19 ± 1.12°C; CI_DIfference_ 0.02–0.87; *p* < 0.05; *t*[35] = 2.11; *η* ^2^ = 0.11), and EXTREME (1.12 ± 0.70°C; CI_DIfference_ 0.01–0.73; *p* < 0.05; *t*[35] = 2.11; *η* ^2^ = 0.11).

## 4 DISCUSSION

Mean CV was higher in MILD compared to both VERY HOT and EXTREME, while there was no effect of heat stress on *D*′. ∆Temp was sensitive to heat stress and elevated in VERY HOT and EXTREME.

Supra‐CV exercise depletes finite fuel sources, drives V̇O_2_ to its maximum, and elicits accumulation of H^+^, ADP, La^−^, and Pi which contribute to exercise intolerance (Poole et al., 2016). High heat stress increases cardiovascular strain with associated impaired muscle activation, fluid and electrolyte (Na^+^/Cl^−^) loss, and reduces aerobic capacity (Armstrong et al., 2007; Lafrenz et al., 2008; Periard et al., 2011; Viveiros et al., 2012). During prolonged high intensity exercise in hot conditions, maximal voluntary contractions are impaired even though muscle force remains unchanged when compared to low heat stress (Nybo et al., 2014; Nybo & Nielsen, 2001). Together, these data indicate hyperthermia‐induced central fatigue, resulting in lowered muscle activation as well as increased accumulation of fatigue‐related metabolites and perceptions of exertion (Nybo et al., 2014, Nybo & Nielsen, 2001). In our experiments, heat stress as little as 38°C negatively affected CV as observed through mild changes in tympanic temperature while *D*′ was unaffected. Thus, even in middle distance and endurance events that are typically <30 min when fluid loss is not the primary concern (Periard et al., 2017), the increased cardiovascular strain, impaired muscle activation, or increased perception of exertion may reduce exercise tolerance. Each of the variables discussed above may have played a role, but without mechanistic measurements we can only speculate on the mechanisms for reduced tolerance in our laboratory experiment.

The most similar experiments we introduced above were designs using the 3 min all‐out cycling test following hot water immersion or more traditional heat stress during exercise (Kaiser et al., 2021; Kuo et al., 2021). In the case of heat stress during the 3 min test, the HI was 43.5°C versus our most extreme condition at 55°C HI (Kuo et al., 2021). Interestingly, the reduction in CV in our design at was similar at an ~4% reduction at 38°C heat stress and a slightly larger effect size at 55°C HI. We did not have core temperature measurements to compare to the preheating study in which core temperature was carefully elevated to ≥38.5°C (Kaiser et al., 2021). In either case, the format of the 3 min test is very different to using 4 separate constant power bouts to intolerance (Poole et al., 2016). It is more likely that heat stress accumulates during the longer duration bouts.

Our experiment and others have reported no change in the curvature constant with heat stress (Kaiser et al., 2021, Kuo et al., 2021). We therefore are unable to add anything new to the literature here. The nature of the curvature constant is elusive and controversial. The parameter exhibits characteristics to support and refute a representation of energy storage mechanisms, dynamics of intramuscular fatigue‐related metabolites, and even conflicting views on whether its source is exclusively peripheral in muscle or includes central components (Poole et al., 2016). It is also worth noting that CV and *D*′ necessarily interact and this is a clear weakness of the two‐parameter model. If the curvature constant is heavily weighted toward a nonoxidative muscle energy storage mechanism, it stands to reason that acute heat stress would be of little consequence.

### 4.1 Limitations

The measurements do conform to the hyperbolic velocity‐duration model, and we feel as though the performances were indicative of true exercise tolerance. However, *D*′ interacts with CV and this is an important limitation in the model. For example, hyperoxia and increased aerobic fitness improve CV, or power, which often results in a reduced *D*′ (Gaesser & Wilson, 1988; Poole et al., 2016; Vanhatalo et al., 2008). Therefore, a reduced CV with heat stress may result in elevated *D*′, which we did not observe. Nonetheless, the interaction of the two parameters makes for problematic interpretation of each. Our experimental design also requires “best guess” particularly when assigning velocities during the first and second constant velocity tests. Without an estimation of the velocity–duration relationship in a volunteer, this is an imperfect iterative process. However, we feel this is relatively inconsequential as the goal is simply a range of tolerance times in order to adequately characterize the hyperbolic relationship. Possibly more concerning is that we are unable to give 24 h of recovery between every exercise bout. We did this to keep the number of laboratory visits reasonable for our volunteers. Counterbalancing the bout order is some mitigation, but the 45 min of recovery is not sufficient to eliminate all fatigue from that second bout of each experimental day.

## 5 CONCLUSIONS

Heat indices at or above 38°C negatively affected the velocity–duration relationship in amateur competitive/recreational runners. Thus, even during relatively short events, such as middle‐distance running, where fluid loss and cardiovascular strain are not typically of great concern, heat stress can negatively impact performance.

## FUNDING INFORMATION

No funding information provided.

## CONFLICT OF INTEREST STATEMENT

We have no conflicts of interest to declare.

## ETHICS STATEMENT

The study protocol was approved by the San Diego State University Institutional Review Board.
